# Subinhibitory Concentrations of Rifampicin Synergize with Linezolid to Delay Resistance Evolution in Clinical Methicillin-Resistant Staphylococcus Aureus

**DOI:** 10.3390/microorganisms14061310

**Published:** 2026-06-11

**Authors:** Chunhua Peng, Lu Lai, Chuanwei Zhang, Menglin Hu, Yalong Qi, Fangrui Liang, Ziyan Chen, Sailan Wang, Xiaohui Huang

**Affiliations:** 1 Department of Basic and Clinical Pharmacology, School of Pharmaceutical Sciences, Anhui Medical University, Hefei 230032, China; 2445010921@stu.ahmu.edu.cn (C.P.); 2445011048@stu.ahmu.edu.cn (L.L.); 2545010992@stu.ahmu.edu.cn (C.Z.); 2545011090@stu.ahmu.edu.cn (M.H.); 2545011082@stu.ahmu.edu.cn (Y.Q.); 2445011014@stu.ahmu.edu.cn (F.L.); 2345010959@stu.ahmu.edu.cn (Z.C.); 2345011014@stu.ahmu.edu.cn (S.W.); 2 Anhui Province Key Laboratory of Major Autoimmune Diseases, School of Pharmaceutical Sciences, Anhui Institute of Innovative Drugs, Anhui Medical University, Hefei 230032, China

**Keywords:** drug resistance, in vitro antibacterial activity, linezolid, MRSA, resistance gene, rifampicin

## Abstract

Methicillin-resistant *Staphylococcus aureus* (MRSA) is a multidrug-resistant pathogen. Long-term clinical use of linezolid readily induces bacterial resistance in MRSA. This study explored resistance evolution and related mechanisms of MRSA to linezolid under subinhibitory concentrations of rifampicin, as well as the antibacterial activity and anti-resistance potential of the combination. Synergistic effects were confirmed via the broth microdilution method, checkerboard method, and time-kill curve assay. The mutant prevention concentration (MPC) was determined to assess suppression of resistant mutant enrichment. We used a 28-day adaptive evolution model and compared resistance dynamics between linezolid monotherapy and its combination with subinhibitory concentrations of rifampicin. We analyzed the growth characteristics, biofilm formation, virulence phenotypes, and resistance-related mutations of induced strains. The combination exerted synergistic or additive effects, reducing the MPC of linezolid, narrowing the mutant selection window, and delaying resistance development. Strains induced by the combination exhibited slower growth, a greater reduction in biofilm formation, and significantly lower hemolytic activity and attenuated in vivo virulence in the *Galleria mellonella* infection model. Sanger sequencing revealed specific mutations in the 23S rRNA gene and the ribosomal protein gene (*rplC*). Linezolid combined with rifampicin synergistically suppresses resistant mutant enrichment and delays resistance evolution, providing experimental support for optimizing anti-MRSA therapeutic regimens.

## 1. Introduction

Methicillin-resistant *Staphylococcus aureus* (MRSA) is highly resistant to β-lactam antibiotics and exhibits strong environmental adaptability and colonization ability. It can widely colonize human skin, mucous membranes, and the respiratory tract, causing various severe infections, such as wound and soft tissue infections, pulmonary infections, systemic bloodstream infections, and infective endocarditis. Compared with methicillin-susceptible *Staphylococcus aureus* (MSSA), MRSA infections are more difficult to treat and have a higher recurrence rate, making it one of the core pathogens of clinical nosocomial infections [1,2]. In recent years, with the long-term overuse of antibiotics in clinical and community settings, the drug resistance spectrum of MRSA has continued to expand. It is not only completely resistant to β-lactam drugs but also gradually develops resistance to traditional first-line drugs such as vancomycin and linezolid. This multidrug resistance phenotype has severely limited anti-infective treatment options, posing an urgent clinical challenge [3,4,5]. Combination antimicrobial therapy can broaden the antibacterial spectrum, enhance bactericidal activity, and effectively delay resistance development. Nevertheless, frequent use of combination antibiotics carries the risk of inducing resistance in microorganisms, making it an important yet carefully balanced research direction for combating multidrug-resistant bacterial infections [6].

Linezolid is the first oxazolidinone antimicrobial agent used clinically. Due to its unique mechanism of action on the 50S ribosomal subunit and lack of cross-resistance with other antibiotic classes, linezolid has become a first-line drug for multidrug-resistant MRSA infections [7,8,9]. However, long-term use is associated with side effects such as thrombocytopenia and gastrointestinal discomfort, and the number of clinically resistant strains has been increasing annually, seriously compromising its therapeutic efficacy. Rifampicin is a broad-spectrum antimicrobial agent that exerts bactericidal effects by inhibiting bacterial RNA synthesis and can penetrate biofilms to achieve effective intracellular concentrations. However, rifampicin monotherapy rapidly drives the emergence of bacterial resistance, so it is often combined with other antimicrobial agents [10,11,12,13]. Current studies have confirmed that the linezolid-rifampicin combination is effective against MRSA-related infections [14,15,16], but there is still a lack of relevant research on the specific characteristics and molecular regulatory mechanisms of MRSA resistance evolution to linezolid under the intervention of subinhibitory concentrations of rifampicin.

Previous reports have indicated that resistance mechanisms of MRSA to linezolid primarily involve three aspects: (1) mutations in ribosome-related genes (e.g., 23S rRNA, *rplC*, *rplD*) impair linezolid binding to the ribosome [7,17,18,19]. (2) horizontal acquisition of resistance determinants such as *cfr*, *optrA*, and *poxtA* can also confer resistance [20,21,22]. Among these, the *cfr* gene encodes a methyltransferase that modifies the bacterial ribosome, thereby conferring resistance to linezolid. (3) biofilm formation and efflux pump expression contribute to resistance by promoting the efflux of linezolid to reduce intracellular drug accumulation [9,23,24]. Among these, ribosome-related gene mutations are the most important and common mechanisms in clinical resistant strains [25,26]. Given the above background, this study used linezolid combined with subinhibitory concentrations of rifampicin. On the one hand, the synergistic effect inhibits bacterial proliferation and reduces the probability of resistance mutations; on the other hand, subinhibitory concentrations of rifampicin provide mild selective pressure to construct an MRSA adaptive evolution model, aiming to explore the resistance evolution rules, phenotypic characteristics, and molecular regulatory mechanisms of MRSA to linezolid. This study is expected to provide experimental evidence and theoretical support for optimizing combination therapy, delaying MRSA resistance, and improving infection prevention and control strategies.

## 2. Materials and Methods

### 2.1. Bacterial Isolates

In this study, three clinical isolates of methicillin-resistant *Staphylococcus aureus* (designated as strains 690, 692, and 706) were obtained from the First Affiliated Hospital of Anhui Medical University. The reference strain ATCC 43300 was obtained from the Anhui Provincial Antimicrobial Resistance Surveillance Center. All tested strains were identified and re-evaluated for drug resistance using the VITEK-2 automated microbial analysis system (bioMérieux, Marcy l’Étoile, France) to verify strain purity and the accuracy of resistance phenotypes.

### 2.2. Antimicrobials and Culture Media

Linezolid and rifampicin (China Food and Drug Administration, Beijing, China), Mueller-Hinton agar (MHA) (Oxoid, UK), and Mueller-Hinton broth (MHB) (Qingdao Haibo Biotechnology Co., Ltd., Qingdao, China) were used in this study.

### 2.3. Antimicrobial Susceptibility Testing

We used the broth microdilution method following the Clinical and Laboratory Standards Institute (CLSI, 2022) guidelines to assess the MICs of linezolid and rifampicin for each test strain. Strain ATCC 43300 served as the quality control. The bacterial suspension was adjusted to a density of 5 × 10^5^ CFU/mL. After inoculation, plates were placed in a 37 °C incubator for 18–24 h. The MIC refers to the lowest drug concentration that inhibits visible bacterial growth. Each experiment was carried out three times to ensure reproducibility.

### 2.4. Checkerboard Assay

To evaluate how linezolid and rifampicin interact in vitro, we employed the checkerboard broth microdilution technique [27]. Drug concentrations for each strain were set across a range of 1/16 × MIC to 2 × MIC, based on the predetermined MICs of each strain. A bacterial suspension with a final density of 5 × 10^5^ CFU/mL was added to each well. After the plates were incubated at 37 °C for 24 h, visible turbidity was examined to determine whether bacterial growth was prevented. Each test was performed in triplicate. The fractional inhibitory concentration index (FICI) was used to characterize the drug–drug interaction, calculated as: FICI = (combined linezolid MIC/linezolid alone MIC) + (combined rifampicin MIC/rifampicin alone MIC). The FICI interpretation criteria were defined as follows: FICI ≤ 0.5 indicates synergy; 0.5 < FICI ≤ 1 indicates additivity; 1 < FICI ≤ 4 indicates no interaction; and FICI > 4 indicates antagonism.

### 2.5. Time-Kill Curve Assay

Time-kill curves were used to further evaluate the 24 h in vitro antibacterial activity of linezolid and rifampicin alone and in combination. Based on prior MIC results, three drug treatment groups were established. The bacterial suspension was adjusted to 5 × 10^5^ CFU/mL. The groups were as follows: 1/2 × MIC linezolid alone, 1/2 × MIC rifampicin alone, and 1/2 × MIC linezolid plus 1/2 × MIC rifampicin. All samples were incubated in a constant-temperature shaker. Samples were collected at 0, 2, 4, 8, 12, and 24 h, and colony counts were determined by the spot plating technique. Each experiment was repeated three times to ensure reproducibility. Additivity was defined as a 1–2 log_10_ CFU/mL reduction by the drug combination compared with the most active single agent, while synergy was defined as a ≥2 log_10_ CFU/mL reduction [28].

### 2.6. Mutant Prevention Concentration

To conceptually validate the pharmacological impact of combination therapy on narrowing the mutant selection window (MSW), the MPC of linezolid was determined in the presence of rifampicin. We determined the mutant prevention concentration (MPC) of linezolid alone and in combination with 1 × MIC rifampicin using the agar dilution technique as described in reference [29]. For the monotherapy group, we prepared a series of linezolid solutions ranging from 0.5 × MIC to 8 × MIC by two-fold serial dilution. These solutions were then mixed with sterilized MH agar to prepare drug-containing plates. For the combination group, a series of linezolid solutions (concentrations from 0.5 × MIC to 8 × MIC) was mixed with an equal volume of 1 × MIC rifampicin to prepare combined drug-containing plates. The bacterial culture was concentrated to 10^10^ CFU/mL. A 100 μL aliquot was evenly spread onto each drug-containing plate. Plates were incubated without shaking at 37 °C for 24, 48, and 72 h. The MPC was defined as the lowest drug concentration that allowed no visible colony growth. All tests were performed in three independent replicates. The selection index (SI = MPC/MIC) was used to evaluate the width of the mutant selection window (MSW). An SI value of ≤1 indicates a closed MSW. A lower SI value represents a narrower MSW and a lower risk of resistant mutant enrichment.

### 2.7. Adaptive Laboratory Evolution Assay

A 28-day continuous drug administration and passage method, as described in the literature [30], was used to establish an adaptive evolution model of MRSA resistance to linezolid. The original parental strain (without drug exposure) was designated as the wild-type strain (WT). Two groups were set up: the linezolid monotherapy induction group and the linezolid combined with rifampicin induction group, with 3 parallel replicates in each group. The initial inoculation concentration of the bacterial suspension was 1.5 × 10^6^ CFU/mL. For the monotherapy group, the initial induction concentration was 1/4 × MIC linezolid, which was then gradually increased to 1/2 × MIC, 1 × MIC, 2 × MIC, and 4 × MIC. After 28 days of adaptive evolution, the strains induced by linezolid alone were designated as linezolid-induced strain (LI), and those induced by linezolid combined with rifampicin were designated as linezolid-rifampicin combination-induced strain (LRI). For the combination group, the linezolid concentration gradient was the same as that in the monotherapy group, while rifampicin was maintained at a constant subinhibitory concentration of 1/2 × MIC throughout the experiment to provide a low inhibitory pressure. During daily passage, cultures with visible turbidity were collected and diluted at a ratio of 1:100 into MHB medium containing the corresponding drug concentration, followed by incubation at 37 °C with shaking at 220 rpm. Bacterial growth was determined by visual observation of the turbidity of the bacterial suspension: if no turbidity was observed, the culture was discarded, and the viable bacterial suspension from the previous generation was resuscitated and re-inoculated; if turbidity was observed, passage was continued. Each drug concentration gradient was continuously passaged for 7 days; if no growth was observed at an increased concentration, the induction was returned to the previous concentration, we proceeded to the next concentration only after stable growth was achieved. Following each bacterial passage, the broth microdilution approach was used to ascertain the MIC of linezolid against the induced strains, with visual reading used to determine the outcomes. The concentration that showed no observable bacterial growth was recorded as the MIC. Each test was conducted in triplicate, and the median value was selected as the final MIC result. Resistance was considered a four-fold or higher increase in the MIC of the induced strain relative to that of the original parent strain [31].

### 2.8. Cross-Resistance Assessment

To assess whether induced strains develop cross-resistance, we followed the CLSI 2022 guidelines and used the broth microdilution technique to determine the MIC values of clinically commonly used antibiotics (vancomycin, daptomycin, tigecycline) and rifampicin against the induced resistant strains. This allowed us to assess the risk of cross-resistance. All experiments were performed three times. The results were compared with those of the wild-type strain.

### 2.9. Growth Kinetics Analysis

The overnight cultures of WT, LI, and LRI were diluted with MHB to 10^6^ CFU/mL. These diluted cultures were incubated at 37 °C with constant shaking at 220 rpm. At hourly intervals for 24 h, aliquots were collected, and optical density at 600 nm (OD600) was measured. Growth curves were plotted for each strain group, with culture time (h) on the horizontal axis and the measured OD600 values on the vertical axis.

### 2.10. Biofilm Formation Assay

A modified microtiter plate approach, as described previously [32], was used to quantitatively evaluate biofilm formation capacities of WT, LI, and LRI. In brief, 0.2 mL of bacterial suspension with a final density of 1 × 10^7^ CFU/mL was dispensed into each well of a 96-well microtiter plate (Labjoy Biotechnology, Beijing, China). Pure MHB was used as a blank control to eliminate background interference. The plates were then incubated at 37 °C for 48 h to permit biofilm formation. Subsequently, the culture fluid was removed, and each well was gently rinsed three times with PBS. After the plates were air-dried, the adherent biofilms were fixed by adding methanol for 15 min, followed by another PBS rinse and a second air-drying step. Subsequently, the samples were stained with 0.1% crystal violet for 20 min under dark conditions. After washing away the unbound stain, we dissolved the biofilm-attached crystal violet with absolute ethanol for 30 min. Absorbance at 590 nm (OD590) was then read on a plate reader, permitting comparison of the biofilm-forming ability among strains. All experiments were independently repeated three times, each in triplicate.

### 2.11. Measurement of Hemolytic Activity

To evaluate the changes in hemolytic activity, we performed the sheep red blood cell hemolysis assay using the WT, LI, and LRI derived from strain ATCC 43300 and clinical strains 690 and 706. Single colonies of the parental and induced strains were transferred to 3 mL of MHB broth. The cultures were shaken at 220 rpm and incubated at 37 °C until they reached the mid-log phase. A sterile loop was then used to pick up the bacterial suspension and streak it onto 5% defibrinated sheep blood agar plates. The streaking was performed in a single line to ensure isolated colonies grew. Three replicate plates were prepared for each group. These plates were kept upside down during incubation at 37 °C for 16 to 20 h. The resulting hemolytic patterns were assessed visually and categorized into three types: β (clear zones around colonies), α (greenish discoloration), or γ (no visible change).

### 2.12. Galleria mellonella Virulence Assay

We used the *Galleria mellonella* larval infection model to further investigate virulence changes in WT, LI, and LRI derived from strain 690. *Galleria mellonella* (greater wax moth) larvae, each weighing approximately 250 mg, were obtained from Henan Keyun Biological Pesticide Co., Ltd. (Zhengzhou, Henan, China). Each group of 10 larvae was infected via microinjection: 10 μL of bacterial suspension (containing 1.5 × 10^7^ CFU) was injected into the hemocoel at the distal part of the left hind leg. The blank control group received an injection of 10 μL of sterile 0.9% NaCl solution. All infected larvae were incubated at 37 °C and monitored daily for 7 days. Death was recorded when a larva showed no response to gentle mechanical stimulation. Survival curves were plotted, and the experiment was repeated independently three times to ensure the results were reliable.

### 2.13. Sanger Sequencing

Sanger sequencing was performed on WT, LI, and LRI, with results aligned against the parental strain sequence. PCR was used to screen for resistance-associated genes: 23S rRNA, *rplC*, *rplD*, *mgrA*, and *norA* for linezolid resistance, as well as *rpoB* for rifampicin resistance. Sequence data at both the nucleotide and amino acid levels were examined with BLAST (accessible at https://blast.ncbi.nlm.nih.gov/Blast.cgi, accessed on 26 January 2026) with SnapGene Viewer 8.0.1, allowing for mutation detection and comparison.

### 2.14. Statistical Analysis

All statistical analyses were performed with GraphPad Prism 10.1.2. Three independent replicates were performed for each experiment. Results are presented as the mean ± standard deviation. For biofilm formation data, which involved two factors (strain type and background isolate), two-way analysis of variance (ANOVA) followed by Dunnett’s multiple comparisons test was applied. *p*-values < 0.05 were deemed statistically significant.

## 3. Results

### 3.1. Minimum Inhibitory Concentration (MIC) and Combined Antibacterial Activity

Table 1 presents the minimum inhibitory concentration (MIC) values and fractional inhibitory concentration index (FICI) outcomes for all tested strains. Against the four MRSA isolates, linezolid exhibited MIC values ranging from 2 to 4 mg/L, while rifampicin had an MIC range between 0.008 and 0.016 mg/L. According to the CLSI 2022 guidelines, all test strains were susceptible to both linezolid and rifampicin, with no drug resistance observed. When the two drugs were combined, we observed synergistic effects in 3 of the 4 strains (FICI ≤ 0.5) and additive effects in strain 692 (0.5 < FICI ≤ 1). No antagonistic effects were found in any of the combinations, indicating potent in vitro synergistic antibacterial activity between linezolid and rifampicin.

### 3.2. Time-Kill Curves

**Figure 1:**
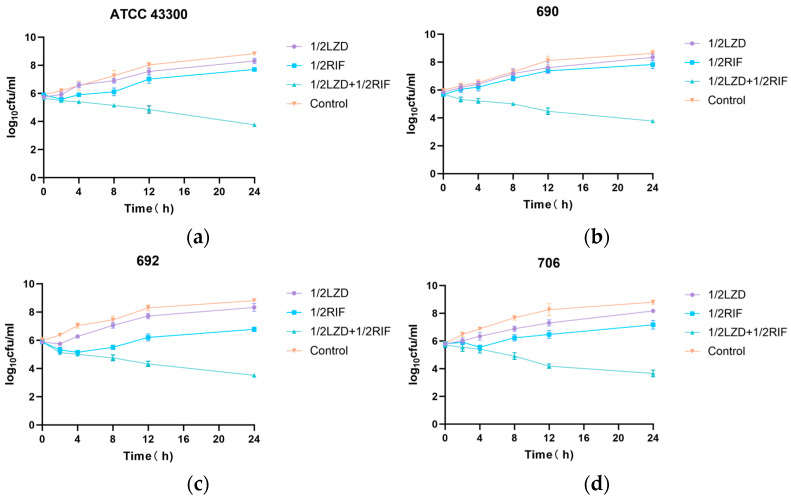
Bactericidal activity against MRSA in time-kill assays with linezolid, rifampicin, and their combination. (**a**) ATCC 43300; (**b**) 690; (**c**) 692; (**d**) 706. Control: no drug; LZD, linezolid; RIF, rifampicin; 1/2, 1/2 × MIC. Data are presented as mean ± SD (error bars indicate SD).

As shown in Figure 1, treatment with 1/2 × MIC linezolid alone exerted only minimal bacteriostatic activity, with bacterial counts comparable to the blank control at all time points. Monotherapy with 1/2 × MIC rifampicin demonstrated superior inhibitory effects compared with linezolid alone, yet it still did not achieve bactericidal activity. In contrast, the combined treatment group exhibited a steady decrease in bacterial density as time progressed, with bactericidal effects that were considerably stronger and more persistent than those of either single-drug treatment. After 24 h, the combination group reduced bacterial load by more than 2 log_10_ CFU/mL compared with rifampicin alone, which was the most effective monotherapy, confirming the synergistic effect.

### 3.3. MPC of Linezolid Alone and in Combination

The MPC and SI values of linezolid alone and in combination with 1 × MIC rifampicin are detailed in Table 2. For linezolid monotherapy, the MPC values across the four tested strains ranged from 4 to 16 mg/L, while their corresponding SI values were between 2 and 4. When linezolid was combined with 1 × MIC rifampicin, the MPC decreased to 2–4 mg/L and the SI was reduced to 1–2. Compared with linezolid alone, combination therapy lowered the MPC and narrowed or closed the mutant selection window for linezolid resistance in MRSA.

### 3.4. Adaptive Laboratory Evolution

**Figure 2:**
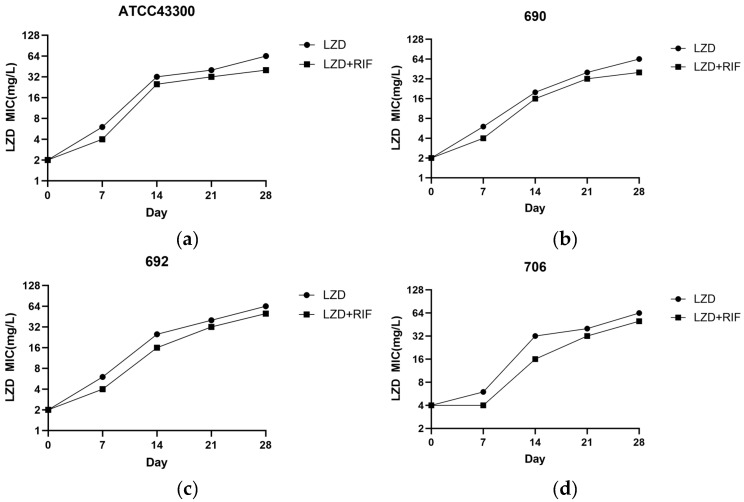
Trend of MIC changes during induction by linezolid alone and in combination with 1/2 × MIC rifampicin. (**a**) ATCC 43300; (**b**) 690; (**c**) 692; (**d**) 706. LZD, Linezolid; RIF, Rifampicin. Data points represent the median MIC values from three independent replicates per exposure.

Figure 2 illustrates the changes in linezolid susceptibility of the four tested strains following 4-week continuous induction with linezolid alone or in combination with subinhibitory concentrations of rifampicin. Throughout the induction period, MIC values of linezolid for all four strains in the combination group were consistently lower than those in the monotherapy group. Combination treatment slowed the rate of MIC elevation and delayed the development of resistance compared with linezolid monotherapy, indicating that subinhibitory concentrations of rifampicin delayed the in vitro evolution of linezolid resistance in MRSA.

### 3.5. Cross-Resistance

The MIC values of induced strains to four anti-MRSA agents (vancomycin, daptomycin, tigecycline) and rifampicin are listed in Table 3. When the MIC values were evaluated against the CLSI 2022 breakpoints, every induced strain remained susceptible, with MICs that matched those of the original parent strain. No signs of cross-resistance emerged from our tests. Collectively, these findings show that MRSA develops resistance to linezolid through a specific pathway, without affecting its response to other common drugs.

### 3.6. Growth Curves

**Figure 3:**
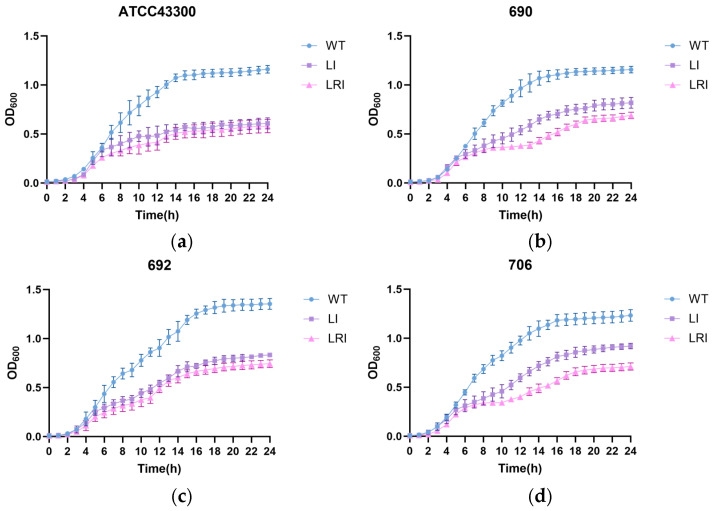
Growth curve trends of linezolid alone-induced and combination-induced strains. (**a**) ATCC 43300; (**b**) 690; (**c**) 692; (**d**) 706. WT, Wild-type strain; LI, Linezolid alone-induced strain; LRI, Linezolid-rifampicin combination-induced strain. Data are presented as mean ± SD (error bars indicate SD).

The growth curves of WT, LI, and LRI are shown in Figure 3. Compared with the WT, LI and LRI grew at a significantly slower rate, with their growth curves remaining below those of the WT throughout. This indicates that antibiotic selection considerably inhibits bacterial growth. Moreover, the LRI grew more slowly than the LI, especially in strains 690 and 706. All four LRI exhibited a typical biphasic growth pattern, a phenotype that suggests resistance evolution may alter bacterial growth behavior. This phenomenon may be related to altered regulation of metabolic pathways or the activation of a stringent response. These findings demonstrate that resistant strains derived under different induction conditions exhibit heterogeneity in their growth phenotypes.

### 3.7. Biofilm Formation

**Figure 4:**
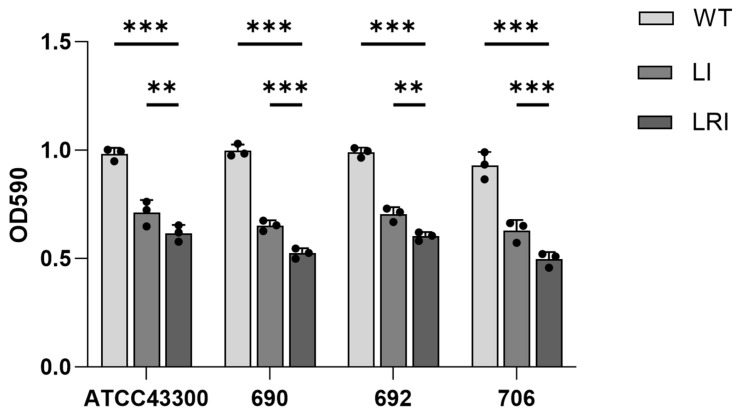
Biofilm formation ability of parental and induced strains. Data are presented as mean ± SD of three independent experiments. ***, *p* ≤ 0.001; **, *p* ≤ 0.01.

Biofilm formation abilities of WT, LI, and LRI are shown in Figure 4. Two-way ANOVA with Dunnett’s test revealed that LRI formed significantly less biofilm than WT in all background isolates (*p* < 0.0001 for all comparisons). Compared with LI, LRI also showed significantly reduced biofilm formation in ATCC 43300 (*p* = 0.010), strain 692 (*p* = 0.006), and the other isolates (*p* < 0.0001 for strains 690 and 706). This indicates that linezolid-induced resistance significantly inhibits MRSA biofilm formation, and the inhibitory effect is further enhanced by the addition of subinhibitory concentrations of rifampicin.

### 3.8. Hemolytic Activity Assay

**Figure 5:**
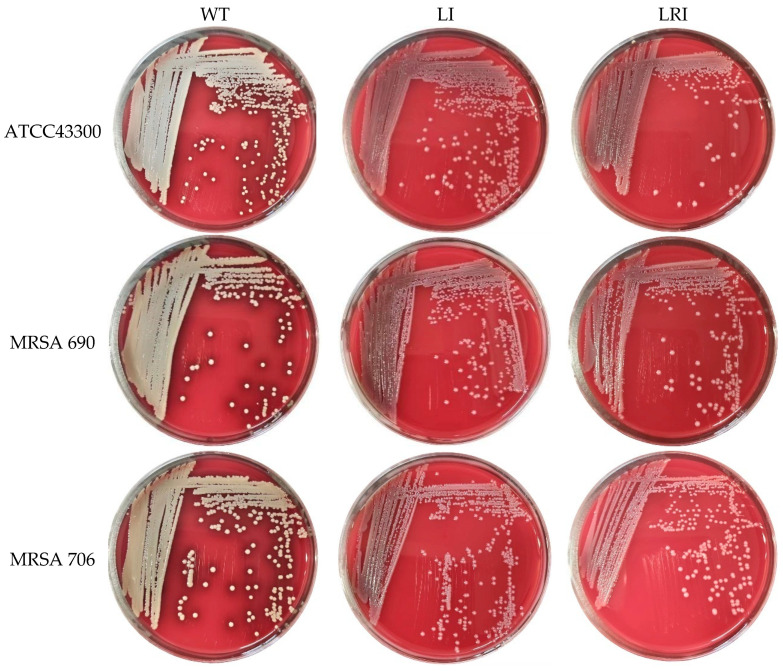
Hemolytic reaction and colony morphology of parental and induced strains.

Based on their superior antibacterial activity in prior assays (MPC, growth curves, and biofilm formation), we selected the WT, LI, and LRI derived from ATCC 43300, 690, and 706 (three of the four initial background strains) for hemolytic activity assessment. The results of hemolytic activity detection for each strain are shown in Figure 5. The WT exhibited typical β-hemolysis, with a clear transparent hemolytic ring visible around the colony, indicating a strong ability to lyse red blood cells. The LI and LRI showed significant changes in hemolytic phenotype, both presenting γ-hemolysis, with no transparent hemolytic ring or color change around the colonies, suggesting that the hemolytic activity of the strains was significantly weakened or completely lost after induction of drug resistance.

### 3.9. Analysis of Virulence of Strains in the Galleria mellonella Infection Model

**Figure 6:**
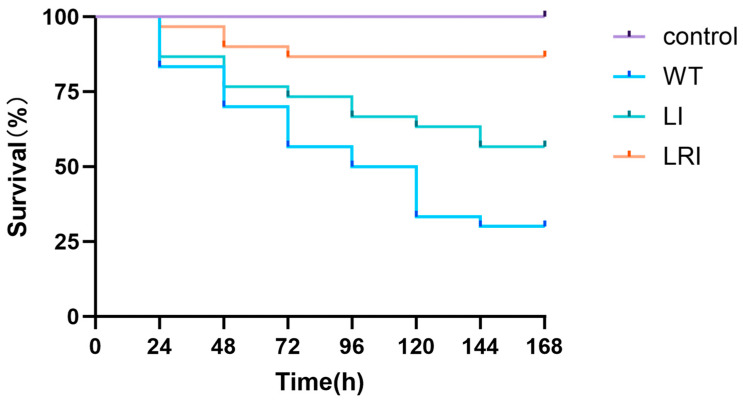
Survival curves of parental and induced strains.

Survival curves from the *Galleria mellonella* infection model are presented in Figure 6. Larval survival rates differed significantly among infection groups. Compared with the WT group, survival was significantly higher in larvae infected with LI and LRI strains. WT showed the lowest survival rate and highest virulence. LRI exhibited the highest survival rate and weakest virulence, while LI showed intermediate survival and virulence. These results demonstrate that linezolid-induced resistance reduces the in vivo pathogenicity of MRSA, and combination with rifampicin further attenuates bacterial virulence.

### 3.10. PCR Amplification and Sequencing

The three strains (ATCC 43300, 690, and 706) along with the parent strain were subjected to Sanger sequencing to detect resistance-associated gene mutations, including 23S rRNA, *rplC*, *rplD*, *rpoB*, *mgrA*, and *norA*. No mutations were detected in *rplD*, *mgrA*, or *norA* in any strain. No target gene mutations were identified in parental strains. Both LI and LRI carried 39 identical mutations in 23S rRNA, 21 (53.85%) of which were located within the peptidyltransferase center (PTC). Typical resistance mutations, including G2576T, G2447T, and T2500A, were not identified in any of the tested strains. Within the *rplC* gene, a serine deletion at position 145 was found solely in the ATCC 43300-LI strain. All other induced strains harbored the G455A mutation, which caused a Gly152Asp amino acid substitution in the ribosomal protein L3. Regarding the *rpoB* gene, the G523A mutation (responsible for an Asp471Asn substitution in the RNA polymerase β-subunit) was detected only in strains induced by the combined treatment, and was not present in the parental strain or those induced by monotherapy. Consistent with cross-susceptibility findings, this mutation did not lead to rifampicin resistance. Table 4 provides a comprehensive summary of the detailed mutation profiles for all strains.

## 4. Discussion

In this study, checkerboard and time-kill assays showed that combining linezolid with rifampicin produced either synergistic or additive effects against all tested strains, with no antagonism observed. This finding is consistent with a previous study [14], confirming that the combination has a potent in vitro antibacterial effect against MRSA. The combined therapy enhanced the inhibitory effect of linezolid alone, providing experimental support for its clinical use.

Bacterial resistance evolution is a key concern in clinical treatment. To understand how combination therapy affects linezolid resistance in MRSA, we combined mutant prevention concentration (MPC) testing with in vitro experimental evolution. The MPC tests showed that the combination significantly reduced both the MPC and the selection index (SI) of linezolid, effectively narrowing the mutant selection window (MSW). A narrower MSW is known to be more effective at limiting the emergence of resistant mutants [33,34]. The MPC assay was performed using rifampicin at 1 × MIC to obtain a stable pharmacological demonstration of MSW closure, whereas the subsequent adaptive evolution experiment was designed to simulate a clinically relevant scenario of subinhibitory drug exposure; therefore, rifampicin was used at 1/2 × MIC. Consistent with these findings, a 28-day in vitro serial passage experiment further confirmed that, compared with linezolid alone, adding a subinhibitory concentration (1/2 × MIC) of rifampicin significantly slowed the rise in linezolid MIC values. Throughout the induction period, MIC levels in the combination group remained consistently lower than those in the monotherapy group. This demonstrates that subinhibitory concentrations of rifampicin delay the development of linezolid resistance in MRSA. Collectively, these two lines of evidence indicate that rifampicin combination therapy suppresses linezolid resistance in MRSA through two key mechanisms: narrowing the resistance selection window and slowing the pace of resistance evolution. We speculate that the mechanism may involve, on the one hand, subinhibitory concentrations of rifampicin can interfere with the auxiliary pathway of bacterial protein synthesis by inhibiting bacterial RNA polymerase, thereby complementing the mechanism of linezolid in blocking the peptidyl transferase center. On the other hand, the combination narrows the mutant selection window and reduces the chance of resistant mutants being enriched, thus preventing resistance at its source [35,36]. In addition, the in vitro serial passage experiment showed no cross-resistance to other antibiotics in the combination-induced strains.

Bacteria commonly acquire resistance through gene mutation or horizontal transfer, often accompanied by reduced fitness, such as slower growth or weakened virulence [37,38,39]. This study examined the phenotypic differences and virulence changes in the induced strains, focusing on their molecular mutation profiles to clarify how the combination regimen regulates resistance. Compared with the wild-type strain, both induced groups showed significant phenotypic changes, and these changes were more pronounced in the combination-induced group. Growth curve analysis revealed that the growth rate of both induced strains was slower than that of the wild-type strain, and the combination-induced strain exhibited a more significant delay and a characteristic biphasic growth pattern not observed in the monotherapy-induced strain. This phenotype may result from two mechanisms. First, ribosomal target mutations in resistant strains interfere with protein synthesis, and additional *rpoB* mutations in the combination-induced strain impair RNA transcription, further enhancing growth inhibition. Second, combined treatment disrupts energy and metabolic pathways, reduces carbon utilization efficiency, and triggers a stringent response upon glucose depletion, leading to growth arrest and the biphasic growth phenotype [40,41,42].

Beyond classical resistance mutations, biofilm formation is among the most common contributors to bacterial antimicrobial resistance [43]. This study explored the role of subinhibitory concentrations of rifampicin combined with linezolid in limiting linezolid resistance development in MRSA from the perspective of biofilm formation. Bacterial biofilms are key determinants of antimicrobial resistance in MRSA and markedly enhance tolerance to environmental stress and antibacterial agents [44,45,46]. In this study, biofilm-forming capacity was significantly lower in combination-induced strains than in monotherapy-induced and parental strains, consistent with previous findings [42]. This observation may be attributed to impaired growth and ribosomal dysfunction in resistant strains, which weaken bacterial adhesion and extracellular matrix production. Subinhibitory concentrations of rifampicin further suppress extracellular secretion, strengthening biofilm inhibition in the combination group and reducing colonization and survival of resistant strains.

Hemolytic activity assays showed that both types of induced strains switched from β-hemolysis to γ-hemolysis with complete loss of hemolytic activity, and virulence attenuation was more evident in combination-induced strains. This may result from reduced hemolysin gene expression due to growth restriction in resistant strains, as bacteria allocate more resources to resistance gene expression than to virulence determinants. Together with the indirect inhibition of virulence pathways by rifampicin, these findings further support the existence of resistance-associated fitness costs. Taken together, the acquisition of antimicrobial resistance is often accompanied by compromised biological traits, including growth and virulence. More significant virulence reduction in combination-induced strains suggests that combination therapy not only delays resistance but also lowers the pathogenic risk of resistant strains, with important implications for clinical control of resistant MRSA infections. Cross-resistance testing further demonstrated that induced resistant strains remained susceptible to clinically used anti-MRSA agents, including vancomycin and daptomycin, indicating high specificity of resistance evolution. These results confirm the clinical safety of this combination regimen, as it does not expand the resistance spectrum of MRSA.

At the molecular mechanistic level, sequencing data from this study revealed that MRSA resistance to linezolid is primarily driven by target mutations in the 23S rRNA gene. More than half of the 39 identified mutation sites were located within the peptidyl transferase center (PTC), which serves as the binding site of linezolid. The PTC lies in the 50S subunit of bacterial ribosomes. It catalyzes peptide bond formation and facilitates protein synthesis, and it also serves as the primary binding site for linezolid [20,47]. Notably, we did not detect the well-known resistance mutations such as G2576T or G2447T, which have been frequently reported in previous studies [9,25,48]. This suggests that the mutational profile of the resistant strains in this study is unique and represents a novel pattern of target mutation. We speculate that combined mutations at multiple sites within the PTC may alter ribosomal conformation to mediate resistance. Therefore, instead of relying on a single canonical mutation site, MRSA develops linezolid resistance through diverse mutational pathways. Notably, we found that LRI strains from multiple independent backgrounds accumulated an identical set of 39 point mutations in the 23S rRNA gene. This convergent evolution indicates that the LZD + RIF combination imposes an extremely strong and unique selective pressure, allowing only one viable mutational path. Critically, the requirement to accumulate all 39 mutations forces MRSA into an evolutionary dead-end. This high fitness cost explains the self-attenuation phenotype of LRI strains, including reduced growth, impaired biofilm formation, attenuated hemolytic activity, and decreased in vivo virulence. Consistent with previous in vivo findings, this unique mutational profile further explains the reliable bactericidal and anti-resistance efficacy of the LZD + RIF combination observed in animal and foreign-body infection models [10,49].

Moreover, mutations in the *rplC* gene were exclusively found in induced resistant isolates. These mutations contribute to structural changes in ribosomal proteins and help support the resistance phenotype [25]. As the target of rifampicin, the *rpoB* gene only carried the Asp471Asn mutation in strains induced by combined medication, and this variation did not lead to rifampicin resistance. This indicates that the mutation is merely a collateral change associated with synergistic drug pressure, rather than a functional mutation responsible for rifampicin resistance. No mutations were detected in *rplD*, *mgrA*, or *norA*, ruling out the possibility that efflux pumps or abnormal regulatory pathways mediate resistance. Collectively, these findings confirm that mutations in 23S rRNA and *rplC* constitute the core resistance mechanism in this study.

At the same time, this study also has certain limitations. Most of the experiments were conducted in vitro, whereas the *Galleria mellonella* model was adopted only for in vivo virulence assessment. Hence, our findings cannot predict the long-term evolution of MRSA resistance under clinical long-term combined medication with subinhibitory concentrations of rifampicin and linezolid. Further validations using mammalian animal models and clinical isolates are required before clinical application guidance. Despite nearly 20 years of preclinical evidence for LZD + RIF synergy, this regimen has not been translated clinically due to pharmacokinetic interactions: rifampicin induces rapid clearance of linezolid, reducing exposure by 35–60% and preventing precise PK/PD control. The number of strains used in the experiment was limited, failing to cover MRSA strains with different genetic backgrounds, which may affect the universality of the results. In addition, only core resistance genes were sequenced and analyzed, and in-depth studies at the transcriptome and proteome levels were not carried out, which failed to further clarify the specific regulatory pathways between gene mutations and phenotypic changes, and the molecular mechanism still needs to be further elucidated. Notably, resistance mechanisms differentially affect virulence. Zhou et al. reported that cfr-mediated linezolid resistance enhances biofilm formation and virulence, whereas our LRI strains exhibited a de-armed phenotype—reduced growth, biofilm impairment, attenuated hemolysis, and lowered in vivo virulence, highlighting the profound impact of resistance pathways on bacterial pathogenicity [50]. Future studies should expand the strain collection and employ multi-omics technologies to further elucidate the molecular mechanisms underlying resistance regulation and the evolutionary network of linezolid resistance in MRSA. In addition, efforts should be directed toward facilitating the clinical application of this combination regimen, thereby providing a more comprehensive basis for delaying the development of MRSA resistance. However, this study confirms that subinhibitory concentrations of rifampicin combined with linezolid synergistically enhance antibacterial activity, delay resistance evolution in MRSA, and reduce the virulence of resistant strains, without inducing cross-resistance. These findings provide an experimental basis for the clinical treatment of multidrug-resistant MRSA infections and for curbing bacterial resistance, highlighting the potential clinical value of this combination regimen.

## 5. Conclusions

Through a series of in vitro pharmacodynamic and serial passaging assays as well as in vivo virulence tests based on the *Galleria mellonella* infection model, this study confirmed that the combination of linezolid and subinhibitory concentrations of rifampicin exerts synergistic bactericidal effects against MRSA, delays resistance evolution, and reduces virulence. However, current technology still requires three key innovations: rapid mutation-targeted detection tools (e.g., for 23S rRNA, *rplC*, *rpoB*), standardized in vitro evolution models for combination regimens, and clinical PK/PD models to optimize dosing regimens. For future perspectives, researchers should prioritize nanoparticle-based co-delivery systems, validation in mammalian infection models, and host immune studies to translate this combination strategy into clinical practice.

## Figures and Tables

**Table 1:** MIC and FICI of linezolid and rifampicin against experimental strains.

Strains	MIC (mg/L)		Combined MIC
	LZD	RIF	FICI
ATCC43300	2	0.008	0.3125
MRSA 690	2	0.016	0.5
MRSA 692	2	0.016	0.625
MRSA 706	4	0.016	0.5

MIC, Minimum inhibitory concentration; LZD, Linezolid; RIF, Rifampicin; FICI, Fractional Inhibitory Concentration Index.

**Table 2:** MPC and SI values of linezolid alone and in combination with 1 × MIC rifampicin against experimental strains.

Strains	MPC (mg/L)		SI	
	Isolates	Combination	Isolates	Combination
ATCC43300	4	2	2	1
MRSA 690	8	2	4	1
MRSA 692	8	4	4	2
MRSA 706	16	4	4	1

MPC, mutant prevention concentration; SI, mutant selection index.

**Table 3:** MICs of Vancomycin, Daptomycin, Tigecycline and Rifampicin Against Parental and Induced Strains.

Strains	MIC (mg/L)			
	Vancomycin	Daptomycin	Tigecycline	Rifampicin
ATCC43300	0.5	1	0.16	0.008
ATCC43300-LI	0.5	1	0.16	0.008
ATCC43300-LRI	0.5	1	0.16	0.008
MRSA 690	0.5	1	0.16	0.016
MRSA 690-LI	0.5	1	0.16	0.016
MRSA 690-LRI	0.5	1	0.16	0.016
MRSA 692	0.5	1	0.16	0.016
MRSA 692-LI	0.5	1	0.16	0.016
MRSA 692-LRI	0.5	1	0.16	0.016
MRSA 706	0.5	1	0.16	0.016
MRSA 706-LI	0.5	1	0.16	0.016
MRSA 706-LRI	0.5	1	0.16	0.016

MIC, Minimum Inhibitory Concentration; LI, Linezolid alone-induced strain; LRI, Linezolid-rifampicin combination-induced strain.

**Table 4:** Results of drug resistance-related gene mutations in parental and induced strains.

Strains	Resistance Gene		
	23S rRNA	*rplC*	*rpoB*
ATCC 43300	-	-	-
ATCC 43300-LI	C2404T, C2461A, C2477T, G2516A, C2534A	Ser145del	-
ATCC 43300-LRI	C2404T, C2461A, C2477T, G2516A, C2534A	455G > A (Gly152Asp)	523G > A (Asp471Asn)
MRSA 690	-	-	-
MRSA 690-LI	C2404T, C2461A, C2477T, G2516A, C2534A	455G > A (Gly152Asp)	-
MRSA 690-LRI	C2404T, C2461A, C2477T, G2516A, C2534A	455G > A (Gly152Asp)	523G > A (Asp471Asn)
MRSA 706	-	-	-
MRSA 706-LI	C2404T, C2461A, C2477T, G2516A, C2534A	455G > A (Gly152Asp)	-
MRSA 706-LRI	C2404T, C2461A, C2477T, G2516A, C2534A	-	523G > A (Asp471Asn)

“-” indicates no mutation. Positive fragments were obtained by PCR amplification of the *rplD*, *mgrA*, and *norA* genes; however, sequencing confirmed no mutations in these genes, so they were not included in the table. The complete list of all mutations in the 23S rRNA gene is provided in Supplementary Table S2.

## Data Availability

The original contributions presented in this study are included in the article/Supplementary Material. Further inquiries can be directed to the corresponding author.

## Supplementary Materials

The following supporting information can be downloaded at: https://www.mdpi.com/article/10.3390/microorganisms14061310/s1, Table S1: Primers used for PCR amplification of resistance-related genes; Table S2: List of 39 mutations identified in the 23S rRNA gene.

## Abbreviations

The following abbreviations are used in this manuscript:

MRSA	Methicillin-resistant *Staphylococcus aureus*
MSSA	Methicillin-susceptible *Staphylococcus aureus*
CLSI	Clinical and Laboratory Standards Institute
MHA	Mueller-Hinton Agar
MHB	Mueller-Hinton Broth
MIC	Minimum Inhibitory Concentration
FICI	Fractional Inhibitory Concentration Index
LZD	Linezolid
RIF	Rifampicin
MPC	Mutant Prevention Concentration
MSW	Mutant Selection Window
SI	Selection Index
PCR	Polymerase Chain Reaction
PBS	Phosphate-Buffered Saline
LI	Linezolid alone-induced strain
LRI	Linezolid-rifampicin combination-induced strain
WT	Wild-type strain
