# Supplementary material for: Subinhibitory Concentrations of Rifampicin Synergize with Linezolid to Delay Resistance Evolution in Clinical Methicillin-Resistant Staphylococcus Aureus
Source: Microorganisms. 2026 Jun 11;14(6):1310. doi: 10.3390/microorganisms14061310 (PMC13304078; doi:10.3390/microorganisms14061310)
Supplement: Supplementary file 1 [file microorganisms-14-01310-s001.zip › microorganisms-4356210-supplementary.pdf]

Table S1. Primers used for PCR amplification of resistance-related genes

Gene name	Primer name	Primer sequence (5′-3′)	Product size (bp)
23S rRNA	23S-rRNA-F	GCGGTCGCCTCCTAAAAG	390
	23S-rRNA-R	ATCCCGGTCCTCTCGTACTA	
rplC	rplC-F	AACCTGATTTAGTTCCGTCTA	822
	rplC-R	GTTGACGCTTTAATGGGCTTA	
rplD	rplD-F	TCGCTTACCTCCTTAATG	1200
	rplD-R	GGTGGAAACACTGTAACTG	
norA	norA-F	ATGAATAAACAGATTTTT	1167
	norA-R	TGAAAGAACAAAATATGTAG	
mgrA	mgrA-F	ATGTCTGATCAACATAAT	444
	mgrA-R	GCATTTGATGAAACAAAGGA	
rpoB	rpoB-F	GTCGTTTACGTTCTGTAGGTG	432
	rpoB-R	TCAACTTTACGATATGGTGTTTC	

Table S2. List of 39 mutations identified in the 23S rRNA gene.

Number	Mutation (E. coli numbering)
1	T2326C
2	A2329T
3	G2338C
4	G2339C
5	C2355G
6	C2356T
7	T2357G
8	A2360G
9	A2361G
10	G2362C
11	G2372T
12	T2373G
13	A2380C
14	C2381A
15	A2384T
16	T2386A
17	C2404T
18	C2406A
19	C2437G
20	C2441T
21	T2458G
22	C2461A
23	T2462C
24	C2464G
25	C2477T
26	G2486C
27	A2488G
28	G2489T
29	G2516A
30	T2530A
31	C2534A

32	T2547A
33	G2548C
34	C2560G
35	A2561T
36	C2568T
37	C2586T
38	C2617T
39	T2619C
